# Leadership Style and Employees' Commitment to Service Quality: An Analysis of the Mediation Pathway *via* Knowledge Sharing

**DOI:** 10.3389/fpsyg.2022.926779

**Published:** 2022-09-12

**Authors:** Munwar Hussain Pahi, Abdul-Halim Abdul-Majid, Samar Fahd, Abdul Rehman Gilal, Bandeh Ali Talpur, Ahmad Waqas, Toni Anwar

**Affiliations:** ^1^School of Business Management, Universiti Utara Malaysia, Sintok, Malaysia; ^2^PAFKIET, Karachi, Pakistan; ^3^School of Business Management and Institute of Zakat Research & Innovation (IPIZ) Universiti Utara Malaysia, Sintok, Malaysia; ^4^Department of Applied Psychology, The Islamia University of Bahawalpur, Bahawalpur, Pakistan; ^5^Department of Computer and Information Sciences, Universiti Teknologi PETRONAS, Seri Iskandar, Malaysia; ^6^School of Computer Science and Statistics, Trinity College Dublin, Dublin, Ireland; ^7^Knight Foundation School of Computing and Information Sciences, Florida International University, Miami, FL, United States

**Keywords:** leadership, commitment to service quality, knowledge share, OCB, job performance, turnover intention, turnover, hospital

## Abstract

Very little attention has been given to understanding the commitment to service quality and desirable outcomes in the hotel industry. This study investigates the impact of directive and participative leadership on the frontline commitment to service quality through the mediation of knowledge sharing. This will eventually help us to generate the employees' commitment to service quality (CSQ) desirable behavior. The survey was distributed to 37 hotels. A total of 235 frontline employees participated in the survey. The study findings show that directive leadership has a negative relationship with CSQ. On the other hand, participative leadership positively influences CSQ. Notably, knowledge sharing mediates between directive leadership, participative leadership, and commitment to service quality. There were positive linkages between commitment to service quality and desirable outcomes, job performance, and organizational citizenship behavior (OCB) and negative relation to turnover intention.

## Introduction

Frontline employee behavior patterns and work attitudes can directly reflect on the customers and impact the performance of a service organization. High service delivery of customer-contact employees plays a vital role in the excellence of an organization because the delivery of service influences the image of the organization in front of customers. Customer-contact employees are directly responsible for one-to-one customer satisfaction, customer service, and service quality related to the high performance of the service organization (Hartline et al., 2003; Fam et al., 2021). Research by De Ruyter and Wetzels (2000); Zou and Migacz (2022), and Hewagama et al. (2019) identified that failure of service by frontline employees is a failure of the service organization. Currently, service organizations face the issue of guaranteeing that border-spanning employees deliver better service to customers who interact with their customers daily and offer service (Bowen et al., 1989). Besides that, many frontline employees find their repetitive responsibilities and roles boring in their daily work schedule. Most of the frontline employees' routine and repetitive acts make it difficult for them to remain committed or engaged in daily scheduled work (Harris and Ogbonna, 2009). Furthermore, frontline employees face frustration or anger from disruptive customers, which creates job dissatisfaction and less commitment toward service delivery. A series of studies identified that committed employees play a vital role in enhancing service delivery and doing extra work beyond what is required of their role (Alexandrov et al., 2007; Yousf and Khurshid, 2021). In their work, Hartline et al. (2003) and Berjaoui and Karami-Akkary (2020), state that committed employees reveal a low level of work stress, put in extra effort, have customer-oriented values, and provide high service to customers. Surprisingly, there is a lack of literature examining these variables and their importance from an employee perspective. Explaining commitment to service quality (CSQ) is very significant in addressing service delivery issues of frontline employees, and the right environment can lead to higher service delivery to customers (Peccei and Rosenthal, 1997; Clark et al., 2009; Pahi et al., 2015). There is enough evidence that shows leadership can enhance the commitment of employees to deliver high service to customers (Clark et al., 2009; Kumar and Krishnaraj, 2018). Several studies indicate that leaders are most suitable for nurturing positive service quality among their subordinates, especially frontline employees. (Clark et al., 2009; Afsar and Umrani, 2019; Ahmad and Umrani, 2019; Pahi et al., 2020). Although different styles of leadership can play a role in motivation (Isaac et al., 2011; Novitasari et al., 2021a,b), performance enhancement (Isaac et al., 2001), service excellency (Graido-Gonzalez et al., 1998; Luu et al., 2019), and commitment of subordinates (Pahi et al., 2015; Ahmed et al., 2020), it is confirmed that leaders have the closest association with the individual commitment of employees. Leaders can engender individual CSQ in service sectors, especially in underdeveloped countries (Imam et al., 2017; Pahi et al., 2020).

Previous studies on CSQ related to management's CSQ at the organization level (Isaac et al., 2001; Little and Dean, 2006; Elmadag et al., 2008; Crick, 2009; Liao et al., 2009; Cheung and To, 2010; Ling et al., 2016). These studies showed that organizational support, climate, underlying elements, and leadership style contribute to employee commitment. To the best of our knowledge, no study has specifically examined the relationship between directive leadership, participative leadership, the moderating role of knowledge sharing, and outcomes of CSQ. Knowledge sharing is considered a moderator, given that knowledge sharing is an underlying mechanism that enhances employees' commitment to delivering high service (Muthusamy, 2009; Saleem and Ambreen, 2011).

## Literature Review

### Employees' Commitment to Service Quality

The concept of commitment as defined by Mowday et al. (1979) and Meyer et al. (1989) is the devotion and willingness of employees to achieve organizational goals. According to Clark et al. (2009), commitment in the service organization is to deliver high service quality and satisfied customers. CSQ has already been recognized in previous literature as an essential determinant of service quality (Ahmed and Parasuraman, 1994; Hartline and Ferrell, 1996; Babakus et al., 2003). Furthermore, CSQ is essential in the workplace (Bass and Stogdill, 1981; Hartline and Ferrell, 1996; Khalid et al., 2016). Previous scholars have documented that employee commitment is crucial for service organizations because these organizations rely on employee commitment and dedication (Tosi and Mero, 2003; Pahi and Hamid, 2015; Pahi et al., 2020).

An individual employee's commitment is expressed through his or her willingness to deliver the best service quality and work beyond the organization's requirements. In other words, employee commitment reflects the level of trust, loyalty, and extra efforts put in by the employee on behalf of the organization. According to Mathieu and Zajac (1990); Peccei and Rosenthal (1997); Oentoro and Popaitoon (2017), and Schwepker et al. (2019), employee commitment refers to employee engagement in a never-ending process and putting extra effort into their work. During the service encounter, leadership and supervisors could not control their employee's actions and behavior toward customers, such as the willingness of frontline employees to satisfy customers and enhance business performance.

Malhotra and Mukherjee (2004) and Hoang et al. (2022) also supported the view that committed employees work harder, put more effort, and commit extra time to work. The findings of Adekola (2012) revealed that employees who are committed to the job will also try to achieve the objectives of the service organization. Commitment and leadership style discussed by previous scholars indicated that leaders always positively influence the commitment of employees (Porter et al., 1976; Clark et al., 2009; Pahi et al., 2015). Furthermore, these studies showed that leadership contributes to a healthy organizational climate, motivation, trust, and high morale, which is how staff members develop a sense of commitment to the organization. Work performance and loyalty to the organization are influenced by high commitment (Mullins and Linehan, 2005). Committed employees are always keen to learn new skills to enhance their performance and provide better service to customers (Morris and Sherman, 1981; Schneider et al., 1998). Also, commitment is the best indicator of performance, job satisfaction, and turnover, which is why leaders pay more attention to commitment.

Path-goal theory provides the conceptual framework for this study (House, 1971). It focuses on directive and participative leadership styles and emphasizes the importance of leadership direction and clarification at the early stages of work and during work to achieve the goal of an organization. The theory explains that leadership guidance is most significant when the employee is unsure about a task or needs guidance during a task which directly influences an employee's commitment and performance (Robin, 2009). It specifies the type of leadership that fits the work environment, thereby influencing employee commitment to productivity in the organization (House and Mitchell, 1974; Northouse, 2013). It also explains how the best leaders develop strategies for knowledge sharing and guidance that assisted employees to enhance their commitment and focus on outputs and productivity (Rafiee and Mohammadi, 2012; Miao et al., 2014). According to the path-goal theory, successful leaders can generate a high level of work by increasing employees' motivation and commitment to the organization through guidance, clarification, and directions. These leaders tend to clarify for employees to help remove obstacles and reduce turnover.

### Directive Leadership

Leadership is known for leading, implementing, planning, guiding, coaching, participating, rewarding, and motivating subordinates. Directive leadership focuses on clarifying responsibilities and tasks to be performed, removing any roadblocks, and explaining what is expected from the employees. Directive leadership falls into the category of autocratic leadership style. This leadership style holds all power and authority and believes that subordinates must follow the rules and regulations (Mullins and Linehan, 2005). According to Bass and Avolio (1993), directive leadership is task-oriented, with centralized authority, persuasive, and manipulative. Mahdi et al. (2014) also explain that directive leadership is associated with direct work process words such as “what to do,” “how to do,” “where,” “when,” and “who should do.” Polston-Murdoch (2013) defines directive leadership as controlled, aggressive, structured, and descriptive inclining subordinates about how to do and what to do. Employees' failure to espouse management values may be a particular problem in service organizations since frontline employees are often required to make decisions and customize service while on the job (Hartline and Ferrell, 1996). A directive leadership style has a significant relationship satisfaction when an employee successfully performs ambiguous tasks. On the other hand, structured and task-oriented work has a negative relationship with commitment (Çokluk and Yilmaz, 2010; Nadarasa and Thuraisingam, 2014). Mahdi et al. (2014) and Islam et al. (2018) explained that directive and supportive leadership positively influence employee commitment. While few other scholars have posited that direct leadership has a negative influence on teachers' commitment due to structural work and one-time direction (Banjarnahor et al., 2018; Firdaus et al., 2019).

Therefore we propose hypothesis

**H1:** *Directive leadership has a negative relationship with commitment to service quality*.

### Participative Leadership

Empowerment was the central idea of top-level management in the 1990s (Collins, 1999). The literature pertaining to that period reported and discussed different concepts relating to empowerment in developed and developing countries. According to Kanter (1983), empowerment was associated with decentralization and delegation. Studies during this period also discussed participative leadership, which included guidance, consulting, delegation, involvement with employees, and evaluation of their ideas, opinions, and recommendations before any important decision or task (Mullins and Linehan, 2005). Participative leaders involve subordinates in discussion or consultation to make reasonable decisions based on consensuses (Bass and Avolio, 1993). With the consultative nature of leadership behavior, employees are more satisfied and committed to their job and organization (Yousef, 2000). Employees who work under a supervisor with a participative leadership style show commitment, involvement, and loyalty (Islam et al., 2018). Employees who are involved in the decision making-process are likely to be more committed to the organization and deliver high service to customers. And participative leadership can induce involvement, loyalty, and commitment among frontline employees. They realize employees are valuable and have the right to take part in any decision; such leaders are likely to enhance employee commitment among frontline employees. Koberg et al. (1999) also found that participative leaders positively associate with empowerment, which enhances employee commitment. Similarly, Park and Shin (2021) argued that participative leaders enhance employees' ability to share innovative ideas and solutions. Such leaders also consider that this was the preferred style of leadership by employees and it was favorable for enhancing the commitment of employees (Bhatti et al., 2019). Therefore, participative leadership style was positively related to commitment to service quality.

Therefore we propose hypothesis

**H2:** *There is a positive relationship between participative leadership and commitment to service quality*.

### Knowledge Sharing as Mediating

In recent times, organizations face difficulties in transferring knowledge to subordinates in the organization. Scholars have claimed that knowledge-sharing behavior affects organizational innovation, learning, and performance in the market (Kumaraswamy and Chitale, 2012; Swift and Hwang, 2013; Nugroho, 2018; Usman et al., 2019a,b). Previous research has documented knowledge sharing with different employee commitments (Liu and Li, 2018), personality traits (Güngöör et al., 2014), employee creativity (Mittal and Dhar, 2015), and sharing attitude (De Vries et al., 2006). Among these various determining factors, leadership has proven to have a very positive and significant influence on knowledge sharing (Politis, 2001; Srivastava et al., 2006). According to Nanoka (2000), leadership plays a vital role in sharing knowledge with subordinates and enhancing employees' understanding to deliver high services to customers. Howell and Avolio (1993) defined that the role of leadership is to cause meaningfulness-related outcomes like motivating employees to go beyond their job requirements. This leadership style provides opportunities for open discussions and shares knowledge regarding new challenges. When leaders have confidence and share knowledge with subordinates, employees will be more willing to put their efforts into innovating and commit to the organization (Nguyen and Mohamed, 2011). High-quality work and services are possible through leadership which facilitates open discussions (Park and Kim, 2018; Wu and Lee, 2020). Cabrera et al. (2006) conducted a study on large multinational organizations and found a positive relationship between commitment and knowledge sharing. Similarly, Han et al. (2014) stated that commitment and knowledge sharing have a positive relationship. Muthusamy (2009) and Saleem and Ambreen, 2011) have also pointed out that knowledge sharing increases commitment. Leadership's knowledge-sharing behavior contributes to organizational learning, and employees can maintain their learning flow (Swift and Hwang, 2013; Baytok et al., 2014; Farooq, 2018). Kim and Cruz (2016) conducted a study on leadership and commitment in the hospitality industry and confirmed that knowledge sharing enhances commitment. Nonetheless, we are still unclear about the equation between participative leadership, directive leadership, knowledge sharing, and commitment to service quality.

Therefore we propose hypotheses

**H3 and H4:** *Knowledge sharing mediates between leadership style (participative and directive) and commitment to service quality*.

### Employees' CSQ and Outcomes

According to Meyer and Herscovitch (2001) and Schwepker and Hartline (2005), employee commitment has been identified as an important factor, and Wright et al. (1997) and Babakus et al. (2003) link it to job-related outcomes. Prior studies indicated that CSQ could have an encouraging influence in containing turnover, high job satisfaction, better job performance, OCB, and loyalty to the organization (Ahmed and Parasuraman, 1994; Parasuraman et al., 1994; Vipraprastha et al., 2018; Soomro and Shah, 2019; Yukongdi and Shrestha, 2020; Umrani et al., 2022). Utilizing Meyer and Herscovitch's (2001) definition of job satisfaction and commitment to the organization, Elmadag et al. (2008) studied the two related constructs and used behavioral theories to link them to CSQ. The study results summarized that frontline employees' CSQ increased commitment, job performance, job satisfaction, and OCB to the firm. The commitment of employees is significant because of its positive impact on outcomes. Similarly, it is reasonable to postulate commitment and job satisfaction as effective outcomes such as job performance, OCB, and turnover intention. The relationship between CSQ and significant individual-level outcomes are important leadership goals that are related to frontline employees' CSQ (Elmadag et al., 2008; Shah et al., 2019). Committed employees enhance job performance, continue impartments, promote effective functioning to satisfy customers, and reduce organization turnover.

Hence, research hypotheses H5, H6, and H7 are proposed.

*CSQ has a positive relationship with job performance*.
*CSQ has a negative relationship with turnover*.
*CSQ has a positive relationship with OCB*.

## Methodology

The study constructs were measured by five-point Likert scales ranging from strongly agree to strongly disagree. CSQ is the main construct of this study and CSQ measurements for this study were adapted from the work of Elmadag (2006); Elmadag et al. (2008). These items were related to the respondents, to assess their emotional attachment to service quality improvement, sense of personal accomplishment by providing high service quality, willingness to make extra efforts, and personal concern about service quality. Job performance most commonly refers to whether a person performs their job well. The five-item scale of Babin and Boles (1996) was used to measure frontline service employees' job performance, such as “satisfy customers” and “service expectations.” OCBs are individual behaviors^1^ that are discretionary, not explicitly recognized by the formal reward system, and that in the aggregate, promote the effective functioning of the organization. Five OCB items were adapted from the work of Graido-Gonzalez et al. (1998). For turnover intentions, a three-item scale was adapted from the work of Van Breukelen et al. (2004) to analyze the intention of employees to leave or stay on the job. For directive leadership, seven items were adapted from Cook et al. (1981) to explain leadership style. For participative leadership, six items were adapted from Arnold et al. (2000). And for knowledge sharing, a four-item scale was adapted from Lin (2007).

## Sample and Data Collection

This study's conceptual framework focused on the frontline employees in hotels. In the current research model, we hypothesized a direct relationship between leadership (directive and participative) and CSQ. Similarly, mediating knowledge sharing was hypothesized. Additionally, this study investigated the outcomes of frontline employees' CSQ such as turnover and OCB. Therefore, a multi-wave design was selected.

Frontline employees play the most important function in a hotel. They help the guests with their arrangements for rooms and resolve issues during their stay in the hotel. These employees have direct customer handling experience, face customers directly, and liaison and communicate with the operating functions and other hotel departments. Frontline employees are most important for hotels because they handle customers directly and their attitude plays a crucial role in the hotels' success.

This study is focused on frontline employees in the hotels. The questionnaires were distributed to 37 hotels in Pakistan's big cities such as Karachi, Lahore, and Islamabad. According to statistical power analysis (Sakai et al., 2018; Hair et al., 2019), the current study required minimum sample size of 240 questionnaires. We decided to administrate 315 questionnaires to frontline employees in 37 hotels. Researchers personally distributed questionnaires with a convenience sampling approach to gather quantitative data. We received 235 filled questionnaires for data analysis.

This study used SmartPLS.3.3 for data analysis to test our hypotheses because it has wide application and is mostly recognized for quantitative data analysis (Chin and Newsted, 1999; Ringle et al., 2012; Hair Jr. et al., 2017). Scholars also explain that smartPLS is a robust method when the objective of the research is to predict relationships among variables or constructs. Additionally, it is an easy-to-handle multi-regression model. Therefore, we considered SmartPLS to be the most appropriate for our study.

## Demographic

This study included demographic variables such as gender, education, marital status, age, and work experience. It included 39.2 percent male employees and 60.8 percent female; the hotel industry had more women than men, and most hotels preferred female frontline staff to handle customers. On marital status, there were 75.1 percent single employees and 29.9 percent married. The unmarried percentage was high because mostly youngers preferred hotel jobs. Regarding the education level of frontline employees, 67.7 percent were degree or diploma holders, 13.3 percent had MBA, 9 percent had other qualifications, and 3.2 percent had completed high school. Concerning the tenure of the employees, those who had completed <5 years were about 75.1 percent, 5–9 years 19.0 percent, 10–15 years 4.8 percent, and over 15 years 1.1 percent. Finally, of the respondents, 15.3 percent were aged between 20–25, 47.6 percent 26–30 years, 26.5 percent 31–35 years, 7.9 percent 36–40 years, 1.6 percent 41–45 years, and 1.1 percent 46–50 years.

## Results

The present study adopted two approaches—structural equation modeling (SEM) and SmartPLS—as recommended by Ringle et al. (2005). In the first step, we assessed the measurement model consisting of item reliability, composite reliability, average variance, and r-square. In step two, we estimated the structural model for the proposed hypothesis testing.

**Figure 1:**
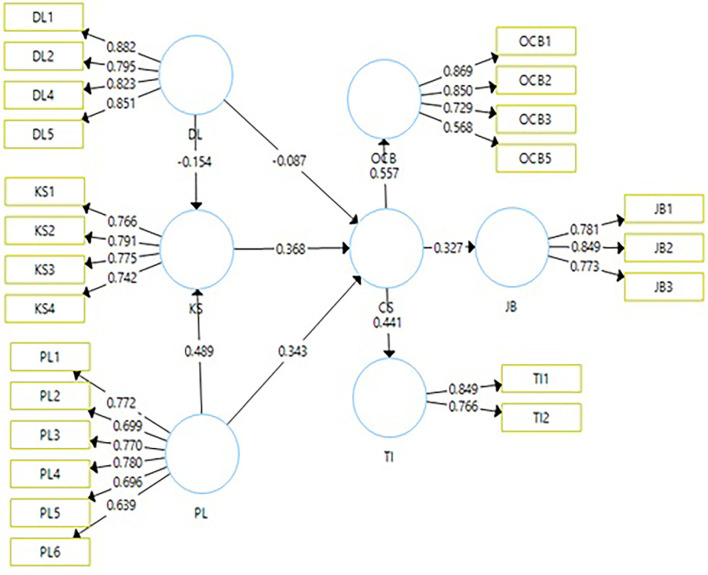
Measurement model.

**Figure 2:**
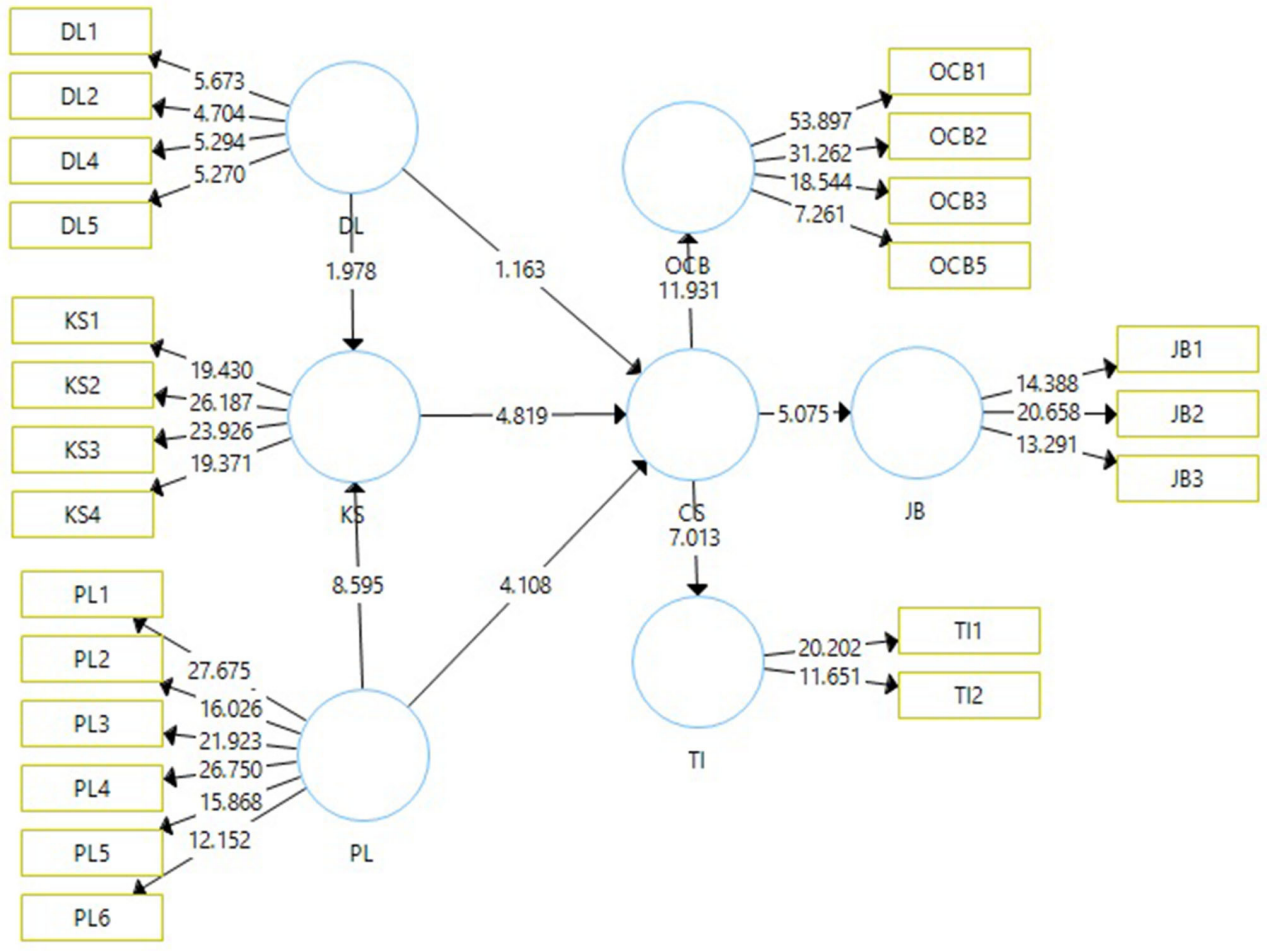
Discriminant validity was assessed as a part of the construct validity, as the multiple items used to measure the same construct should be in agreement, while items between different constructs should be distinct (Campbell and Fiske, 1959).

Data cleaning was undertaken using SPSS to weed out incomplete questionnaires. After cleaning the data, SmartPLS was used to assess the reliability and validity of the questionnaire. The average variance should be 0.5 or above (Hair et al., 2011), and outer loadings above 0.5 are acceptable while values above 0.7 are considered highly satisfactory (Hair et al., 2011, 2014). The results of our tests are shown in Figure 1 and Table 1. Outer loadings ranged from 0.558 to 0.882, indicating the quality of the measurement model was good. The average variance ranged from 0.530 to 0.703 indicating each construct's acceptance. The composite reliability and conventional cutoff limit of the measurement model need to exceed 0.7. As shown in Figure 2 and Tables 1, 2 the composite reliability of our measurement model ranged between 0.790 and 0.904. All constructs met the requirements of the measurement model.

**Table 1:** Measurement model.

**Construct**	**Items**	**Loadings**	**AVE**	**CR**	**R-squr**
Commitment to service quality					
	CS1	0.772	0.579	0.805	0.389
	CS2	0.744			
	CS3	0.766			
directive leadership			0.703	0.904	
	DL1	0.882			
	DL2	0.795			
	DL4	0.823			
job performance	DL5	0.851			
			0.642	0.843	0.107
	JB1	0.781			
	JB2	0.849			
	JB3	0.773			
Knowledge sharing			0.591	0.852	0.256
	KS1	0.766			
	KS2	0.791			
	KS3	0.775			
	KS4	0.742			
Organization behavior			0.583	0.845	0.310
	OCB1	0.869			
	OCB2	0.850			
	OCB3	0.729			
	OCB5	0.568			
Participative leadership			0.530	0.871	
	PL1	0.772			
	PL2	0.699			
	PL3	0.770			
	PL4	0.780			
	PL5	0.696			
	PL6	0.639			
Turnover intention			0.654	0.790	0.195
	TI1	0.849			
	TI2	0.766			

**Table 2:** Discriminatory validity.

	**1**	**2**	**3**	**4**	**5**	**6**	**7**
CS	**0.761**						
DL	−0.121	**0.838**					
JB	0.327	0.111	**0.801**				
KS	0.546	−0.133	0.292	**0.769**			
OCB	0.557	−0.056	0.307	0.586	**0.764**		
PL	0.517	0.042	0.573	0.482	0.627	**0.728**	
TI	0.441	−0.139	0.323	0.560	0.484	0.480	**0.808**

*Bold value indicates the square root of AVE should be greater than the compared reflective loadings of other constructs in a cross-loadings table*.

The second approach, the structural equation model (SEM), was conducted to test the proposed hypotheses. Through SEM, we assessed the impact of directive and participative leadership styles on CSQ, the outcomes of CSQ, and the mediating knowledge sharing between leadership styles and CSQ. The result of the structural model Table 3 and Figure 2 showed that the directive leadership style had a negative influence (DL-CSQ_b = −0.087, *t* = 1.181, *p* = 0.119) on CSQ. H1 accepted. The directive leadership style avoids collaboration and has no clear guidelines, and that behavior style negatively influences frontline employees' CSQ. H2 related to participative leadership style's positive impact (PL-CSQ_B = 0.343, *T* = 4.144, *P* = 0.000) on CSQ. H2 was accepted. The results of the second hypothesis showed in Table 3 and Figure 2 that participative leadership encourages collaboration, improves morale, and free flow of ideas. H3 assessed whether knowledge sharing mediated between directive leadership style and CSQ had a negative relationship (DL-KS-CSQ_b = −0.057, *t* = 1.945, *p* = 0.026). This mediating path negatively influenced CSQ plausibly since directive leadership is unwilling to share knowledge with subordinates. Result of structural model in Table 3 and Figure 2 showed a negative influence on CSQ. H4 assessed whether knowledge sharing mediated between participative leadership style and CSQ had negative relationship (PL-KS-CSQ_b = 0.180, *t* = 4.326, *p* = 0.000). This mediating path showed knowledge sharing positively influenced CSQ because leaders share knowledge with subordinates and allow them to participate in the discussion to present new ideas. The present study investigated the outcomes of CSQ. H5 assessed outcomes of CSQ on job performance, and it was found to be positive (CSQ, JB = *b*, 0.32, *t*, 5.075, pv 0.000) and H6 assessed outcomes of CSQ on OCB, and it was found to be positive too (CSQ, OCB = *b*, 0.557, tv, 11.931, pv 0.000). The results of the study supported the argument that when frontline employees are committed, it would increase employee productivity, improve employee performance, encourage them to do extra beyond requirement, and enhance social interaction between employees. The last hypothesis on turnover had significant negative outcomes of (CSQ, TI_−0.441, *t*, 7.013, *p*_0.000) i.e., committed employees are loyal to the organization and do not intend to leave.

**Table 3:** Structural model.

**Relationships**	**Beta Value**	**(STDEV)**	***T*-value**	***P* values**
DL → CS	−0.087	0.073	1.181	**0.119**
PL → CS	0.343	0.083	4.144	**0.000**
DL → KS → CS	−0.057	−0.056	1.945	**0.026**
PL → KS → CS	0.180	0.179	4.326	**0.000**
CS → JB	0.327	0.064	5.075	**0.000**
CS → OCB	0.557	0.047	11.931	**0.000**
CS → TI	−0.441	0.063	7.013	**0.000**

*Bold value indicates P-value which shows that significance of constructs*.

## Discussion

Leadership styles are essential for enhancing employees' commitment to do better. The frontline employees must be guided and allowed to participate in decision-making so that they understand the impact of the delivery of services at every step; a directive leadership style is unable to do that and therefore has no relationship with employees' CSQ. The results of the current study are in line with Banjarnahor et al. (2018) and Firdaus et al. (2019), that directive leadership focuses on a specific direction, is autocratic in nature, and has less influence on employee commitment. This type of leadership known for being autocratic, limits freedom and avoids any involvement in decision-making, and the leader imposes decisions without any feedback from the employees.

The results of this study confirmed that participative leadership has a significant positive relationship with CSQ. Participative leadership is not only significant but stronger than directive leadership. The results of our study are supported by Dolatabadi and Safa (2010) and Bell and Mjoli (2014). Scholars have explained that participative leadership style plays a significant role in enhancing frontline employees' CSQ and brings positive outcomes. Furthermore, participative leaders enhance motivation, job satisfaction, performance, working conditions, and CSQ of frontline employees (Redshaw, 2000). Participative leadership is also supported by the findings of our study which show that frontline employees need guidance and coaching while delivering service to customers. Participative leaders' timely support inspires and involves frontline employees in decision-making to improve services. Furthermore, the study analyzed the mediation path between directive and participative leadership styles, and CSQ.

The result of path modeling confirmed that knowledge sharing had a significant negative influence between directive leadership and CSQ. The hypothesis was rejected by the results of the study. Our results are in line with the findings of scholars (Dolatabadi and Safa, 2010; Mahdi et al., 2014). which clearly defined that directive leaders are not willing to share knowledge from time to time with frontline employees during service delivery. Our result also confirmed that the autocratic nature of leadership never accepts or allows an employee to take part in decision-making processes and imposes decisions without any feedback. Furthermore, the results indicate that directive leadership is task-oriented authority and avoids sharing knowledge during service delivery to customers.

The results of the path modeling confirmed that knowledge sharing had a significant positive relationship between participative leadership and CSQ. The hypothesis was accepted. A significant and positive relationship between participative and CSQ was established by these results, which are in line with Huang et al. (2010) and Chang et al. (2021). It confirmed that participative leaders share information with employees which directly influences frontline employees' CSQ. Participative leaders are willing to share their knowledge, guide, and coach employees which has a positive influence on their CSQ. Our result explained that the leader's role in sharing knowledge with employees enhances their commitment and encourages them to participate in decision-making processes. Our results are consistent with the arguments of Iqbal et al. (2018), Mohammed and Kamalanabhan (2020), and Sharif et al. (2021) which state participative leadership can increase involvement and commitment among employees through information sharing. These results explain that frontline employees' participation in decision-making is clearly linked to ownership and commitment of employees and stimulates positive attitudes and behavior toward quality service delivery. When leaders share knowledge, guide, and coach employees, it influences their commitment and loyalty.

CSQ is manifest in the positive outcomes of frontline employees' job performance, OCB, and turnover intention. When CSQ is higher than job performance, OCB increases and turnover intention reduces. The result of frontline employees' CSQ and job performance is consistent with the findings of scholars like Babakus et al. (2003) and Ashill et al. (2008) who have argued that CSQ enhances job performance and loyalty in organizations. Our results confirmed that CSQ is a positive indicator of frontline employees' performance. The results of our study identified that the second outcome of CSQ was OCB. CSQ had a positive influence on OCB, especially when the level of frontline employees' CSQ is high, this enhances the morale of employees who then engage in continuous improvement, put in extra effort into satisfying customers, and help and volunteer for extra work. Our results are consistent with the findings of Siregar et al. (2019), Khaskheli et al. (2020), and Sumarsi and Rizal (2022) which demonstrated that frontline employees with higher CSQ accomplish given tasks better and behavior reflects OCB. The path modeling result relating to the third outcome of CSQ and turnover intention showed a significant negative relationship. It indicated that continued enhancement in frontline employees' CSQ would reduce turnover intention. This was consistent with other research (Ashill et al., 2006; Cheung and To, 2010; Karatepe and Karadas, 2012) which also found that frontline employees with high CSQ were less likely to leave the organization than less committed ones. This established that the relationship between frontline employees' CSQ and their job outcomes in a service organization was positive.

The study's conclusion is that the directive leadership style has a significant negative influence on frontline employees' commitment to service quality in Pakistani hotels. Participative leadership has a significant positive influence on the commitment to service quality. Knowledge sharing plays a mediating role between participative and frontline employees' CSQ and is vital to promoting service delivery. Mediating knowledge sharing has a negative relationship between directive leadership and frontline employees' CSQ. The findings also indicated that frontline employees' CSQ positively influences performance outcomes and has a negative relationship with turnover intention. This study builds on prior studies and presents valuable insights for influencing the performance of frontline employees of hotels in the service sector.

This study established that the style of leadership can enhance CSQ in frontline employees. Senior management and leaders could use this as a tool to accomplish CSQ in their organizations. This study offers insight into which leadership style is most important to enhance frontline employees' CSQ and related performance outcomes. The study confirmed that participative leadership is significant in hotel management as compared to directive leadership. Management of the hotel is about assigning the right task to the right leader to motivate and enhance the commitment of employees to deliver quality services to customers. Participative leadership creates an environment where employees are likely to be more engaged in the workplace and try to deliver quality service. This research provides insight to managers about the positive outcomes of CSQ. This study also enhances the understanding of CSQ in frontline employees and how they deal with customers. Besides, the most important implication of this study is to enhance CSQ, reduce turnover, increase job performance, and encourage self-motivation to do extra work to satisfy customers. The hypotheses in our study contribute to the literature that directive leadership has a negative influence, participative leadership has a positive relationship, and knowledge sharing has a mediating role on frontline employees' CSQ. Previous studies on knowledge sharing investigated the directive and participative relationship with CSQ, but the mediating function of knowledge sharing had not been studied yet. This study contributes to hotel industry literature by recognizing the influence of directive and participative leadership styles on frontline employees' CSQ. Previous studies identified CSQ with other variables, but this is the first time a study contributed to linking CSQ and desirable performance outcomes.

## Limitations and Future Research

This study was conducted in a single service industry in Karachi, Sindh, Pakistan. In the study, 72% of respondents were female. The results of the study may not be generalized to other contexts. Future research could expand the scope of research to cover other cities, and other countries, and collect more data. The present study only covers directive and participative leadership styles with particular reference to frontline employees' CSQ. Human resource aspects, such as servant leadership, transaction leadership, delegative leadership, HRM practices, and job satisfaction are excluded from this study. Future research should include other aspects of human resources. This study's CSQ outcomes are limited to only turnover, job performance, and OCB. However, other outcomes of CSQ can be investigated to get a clearer understanding of CSQ. This study assessed respondents' perception of quality and delivery with leadership style involvement. This study is based on a cross-sectional design. Further, research may be conducted with longitudinal data from the same sector.

## Data Availability Statement

The original contributions presented in the study are included in the article/supplementary material, further inquiries can be directed to the corresponding author/s.

## Ethics Statement

Ethical review and approval was not required for the study on human participants in accordance with the local legislation and institutional requirements. Written informed consent from the patients/participants or legal guardian/next of kin was not required to participate in this study in accordance with the national legislation and the institutional requirements.

## Author Contributions

MP: conceptualized and wrote introduction. TA: interpretation of data. AW: revised manuscript and editing. BT: literature review. AG: software and data analysis. SF: revising literature and writing the conclusion. A-HA-M: extensively edited the manuscript and wrote the methodology section.

## Conflict of Interest

The authors declare that the research was conducted in the absence of any commercial or financial relationships that could be construed as a potential conflict of interest.

## Publisher's Note

All claims expressed in this article are solely those of the authors and do not necessarily represent those of their affiliated organizations, or those of the publisher, the editors and the reviewers. Any product that may be evaluated in this article, or claim that may be made by its manufacturer, is not guaranteed or endorsed by the publisher.

## Footnotes

^1^Leadership style, which refers to the way leaders behave toward or treat the individuals they are leading Ehrhart (2004), Drucker (1993) indicated that the quality and performance of managers are the key criteria in deciding organizational success.
